# Application of Raman Spectroscopy and Univariate Modelling As a Process Analytical Technology for Cell Therapy Bioprocessing

**DOI:** 10.3389/fmed.2018.00047

**Published:** 2018-03-05

**Authors:** Marc-Olivier Baradez, Daniela Biziato, Enas Hassan, Damian Marshall

**Affiliations:** ^1^Cell and Gene Therapy Catapult, London, United Kingdom

**Keywords:** raman spectroscopy, cell therapy, bioprocessing, PAT, real-time monitoring

## Abstract

Cell therapies offer unquestionable promises for the treatment, and in some cases even the cure, of complex diseases. As we start to see more of these therapies gaining market authorization, attention is turning to the bioprocesses used for their manufacture, in particular the challenge of gaining higher levels of process control to help regulate cell behavior, manage process variability, and deliver product of a consistent quality. Many processes already incorporate the measurement of key markers such as nutrient consumption, metabolite production, and cell concentration, but these are often performed off-line and only at set time points in the process. Having the ability to monitor these markers in real-time using in-line sensors would offer significant advantages, allowing faster decision-making and a finer level of process control. In this study, we use Raman spectroscopy as an in-line optical sensor for bioprocess monitoring of an autologous T-cell immunotherapy model produced in a stirred tank bioreactor system. Using reference datasets generated on a standard bioanalyzer, we develop chemometric models from the Raman spectra for glucose, glutamine, lactate, and ammonia. These chemometric models can accurately monitor donor-specific increases in nutrient consumption and metabolite production as the primary T-cell transition from a recovery phase and begin proliferating. Using a univariate modeling approach, we then show how changes in peak intensity within the Raman spectra can be correlated with cell concentration and viability. These models, which act as surrogate markers, can be used to monitor cell behavior including cell proliferation rates, proliferative capacity, and transition of the cells to a quiescent phenotype. Finally, using the univariate models, we also demonstrate how Raman spectroscopy can be applied for real-time monitoring. The ability to measure these key parameters using an in-line Raman optical sensor makes it possible to have immediate feedback on process performance. This could help significantly improve cell therapy bioprocessing by allowing proactive decision-making based on real-time process data. Going forward, these types of in-line sensors also open up opportunities to improve bioprocesses further through concepts such as adaptive manufacturing.

## Introduction

The past few years has seen significant growth in the cell and gene therapy field with an increasing number of products receiving market authorization and approximately 900 active clinical trials (1). Despite this growth, cell therapy developers still face many challenges. Among these is the need to develop robust manufacturing processes that can accommodate the complexity associated with live cell therapies in order to make products to a consistent quality. A number of recent articles have suggested that the way to address this challenge and mitigate risks during cell therapy manufacturing could be through the development of processes according to the principle of quality by design (QbD) (2, 3). QbD is a risk-based framework for process design which incorporates prior product knowledge with the use of statistically designed experiments, risk analysis, and product characterization. The intent of QbD is to establish acceptable operating envelopes for a manufacturing process within which a product will be made to a consistently high quality. These operating envelopes are established by understanding and measuring the link between the critical process parameters and critical quality attributes of the therapy.

A key enabler of QbD is the implementation of Process Analytical Technologies (PAT) to allow process and quality to be directly measured. PAT is a framework launched by the FDA in 2004 for “*designing, analyzing and controlling the manufacturing process through the measurement of critical quality and performance attributes with the goal of ensuring final product quality”* (4). The aim of PAT is to encourage the adoption of more advanced in-process monitoring approaches, particularly using technologies that permit in-line or at-line analysis of key variables throughout the manufacturing process [reviewed (5)]. Of particular importance are PAT technologies that allow real-time monitoring of a bioreactor system using in-line sensors, as these can provide quality assurance during final manufacture while also allowing systems to remain closed, thereby minimizing the risk of contamination (6). An important potential advantage of PAT for cell therapy manufacture is the provision of process information in a time frame sufficient to allow proactive decision-making. This has the potential to allow a tighter level of control over complex cell therapy manufacturing processes and allow the early detection of poor process performance.

A large number of cell therapies already apply some form of at-line analysis during the manufacturing process, with samples of cells or culture medium removed and analyzed close to the process stream. This is often achieved using immunoassays such as ELISA to measure secreted proteins, florescent flow cytometry to measure cell surface or intracellular markers, or RT-qPCR to measure gene expression. These techniques can provide quantitative information about the expression of their target analytes and their related quality attributes but take several hours to perform and can only realistically be applied at limited number of time points throughout the manufacturing process. Other more rapid at-line techniques are also available for process monitoring. In particular, bioanalyzers are commonly used to monitor markers related to cellular metabolism including consumption of nutrients such as glucose and glutamine and the production of metabolic by-products such as lactate and ammonia. Several at-line bioanalyzer systems are available to measure these important metabolic markers, but still require the removal of a media sample from the culture system. Again this often limits the analysis to set time points during the manufacturing process. In all cases, the time taken to analyze samples using at-line techniques and/or the requirement to remove samples from the culture system means that process decisions are taken retrospectively, reacting to cellular events that could have happened many hours or days before the data becomes available.

PAT using in-line technologies is increasingly being applied to support biopharmaceutical production (7, 8). Traditionally this has been achieved using standard physical sensors to measure parameters within a bioreactor such as pH, dissolved oxygen, temperature, flow rate, and stirrer speed. However, more sophisticated methods are starting to be adopted, including non-invasive optical sensors such as infrared spectroscopy (NIR and MIR) and Raman spectroscopy. These non-destructive technologies can be used in-line to provide simultaneous real-time information about multiple components of the culture environment, including the consumption of nutrients and the production of metabolic waste products. The use of infrared spectroscopies has been widely reported for analysis of samples during bioprocessing (9–11). However, strong interactions with water can mask the signals from target analytes in aqueous systems such as cell culture media. In comparison, Raman spectroscopy measures the amount of light scattered inelastically at different frequencies by molecular vibrations. This produces detailed molecular fingerprints with high chemical specificity which are only weakly affected by interactions with polar molecules such as water. Consequently, the last few years has seen Raman spectroscopy be increasingly applied for process monitoring during biopharmaceutical production using CHO cell lines (12–14). While the culture environment can be more complex during cell therapy manufacture, the potential for using Raman spectroscopy as an in-process optical sensor to monitor real-time changes is potentially very attractive. Furthermore, the application of real-time monitoring can complement the transition toward the use of closed single-use systems for product manufacture which are increasingly used to improve consistency and reduce cost of goods (15).

Over the past few years, a number of commercially available Raman systems have been developed to support pharmaceutical applications. However, Raman spectroscopy is not a plug and read optical sensor technology. Raman probes placed directly into the cell culture medium provide a molecular fingerprint relating to the vibrational spectroscopic information for all the molecular components within the system. Therefore, the spectroscopic data often needs to be modeled using multivariate analysis approaches such as partial least squares (PLS), principal component analysis (PCA), or artificial neural networks (ANN) to extract the maximum amount of relevant information from the spectral data (16, 17). As such Raman probes are often referred to as “soft sensors” as they require this statistical modeling in order to provide univariate or multivariate information in a format similar to common hardware sensor (18). This means that Raman spectroscopy is more applicable to defined manufacturing processes or during process optimization rather than a tool for early process development.

Here, we show the application of Raman spectroscopy for monitoring changes during bioprocessing of a T-cell immunotherapy in a stirred tank bioreactor system. Using chemometric modeling, we demonstrate how multiple markers of metabolic processes can be tracked in real-time with correlation to at-line measurements using both mass spectrometry and bio-profiling techniques. Furthermore, using a non-targeted approach, we show how Raman peaks can be identified which allow real-time label-free tracking of the variability in cell recovery and proliferation from T-cells isolated from multiple donors. These results represent a significant step forward for real-time cell therapy process monitoring and open up new opportunities to improve the consistency of cell therapy manufacture.

## Materials and Methods

### Cell Starting Material

Fresh non-mobilized peripheral blood leukapheresis products from four healthy donors were obtained from HemaCare Corporation. Leukapheresis were collected in HemaCare’s FDA-registered collection centers following cGMP and cGTP collection guidelines from healthy human volunteer donors under IRB approved informed consent. Transportation from collection center to processing site was performed at 4°C (controlled temperature shipment). Leukapheresis products were processed separately within 48 h of collection.

### T-Cell Isolation and Cryopreservation

Unless otherwise stated materials in this section were obtained from Milytenyi Biotech GmbH. T-cells enrichment from leukapheresis material (half collection) was performed using the CliniMACS^®^plus device by positive selection of CD4+/CD8+ cells. Prior to processing, samples were analyzed for total white blood cell concentration and the number of CD4+ and CD8+ T-cell was measured. Next, 20% human serum albumin (HSA) (Biotest) was added to the CliniMACS^®^ PBS/EDTA buffer in a final concentration of 0.5% (w/v). To reduce platelets contents, the leukapheresis material was diluted 3× in CliniMACS^®^ PBS/EDTA buffer and concentrated 20× using the Sepax2 cell processing system (Biosafe). The cell suspension volume was then adjusted to a volume of 95 mL with CliniMACS^®^ PBS/EDTA buffer before adding a mixture of 7.5 mL anti-CD4 and 7.5 mL anti-CD8 magnetic beads. The samples where then incubated at room temperature for 30 min on a rocking platform set at 25 RPM according to manufacturer’s instructions. At the end of the incubation time, 400 mL of CliniMACS^®^ PBS/EDTA/0.5% HSA was added to the cell suspension and was centrifuged at 500 × *g* for 30 min at room temperature. The supernatant was removed and cells were suspended in CliniMACS^®^ PBS/EDTA/0.5% HSA up to a volume of 100 mL. Positive selection of CD4+ and CD8+ cells was then carried out using the CliniMACS Plus system with the Enrichment 1.1 program. At the end of the selection program, samples were taken for purity analysis. The CD4/CD8 positive cells were centrifuged in a 500 mL centrifuge bottle (Corning) at 1,000 × *g* for 15 min at 4°C. The supernatant was removed and the pellet re-suspended in cold CryoStore 10 (Sigma Aldrich), before controlled rate freezing and storage in liquid nitrogen. A CD3 T-cell purity > 90% was achieved in all final products.

### T-Cell Bioprocessing

T-cell activation and expansion was performed in a DASbox Parallel Mini Bioreactor System equipped with Eppendorf BioBLU 300 mL single-use vessels using a fed-batch process. Each vessel was initially filled with 110 mL TexMACS media (Miltenyi Biotech GMbH) supplemented with 5% human serum (SeraLab), then fitted with dissolved oxygen (DO), pH, temperature, and Raman probes, and allowed to equilibrate overnight with stirring at 80 RPM. For the inoculation of the cell culture, frozen T-cells were thawed, washed, and re-suspended at a concentration of 1 × 10^6^ cells/mL in 10 mL of TexMACS media supplemented with 5% human serum, 1:100 dilution of research grade T-Cell TransAct (Miltenyi Biotech GMbH) and 120 U/mL of IL-2 (Proleukin^®^, Novartis). Cells were maintained in the bioreactors for 12 days with addition of TexMACS media supplemented with 5% human serum and IL-2 (120 IU/mL final concentration) on days 2 (90 mL) and 5 (50 mL). A combination of sodium bicarbonate and carbon dioxide was used to control the pH at 7.2 and an overlay of 5% CO_2_/air was used to maintain dissolved oxygen at 90%. In each experiment, T-cells banked from four different donors were cultured in parallel in 4 Eppendorf BioBLU single-use vessels for 12 days. A total of three experiments with the same four T-cell banks were performed.

### Measurement of Cell Metabolism

The bioreactors were sampled daily to measure cell density and viability using the Vi-Cell XR (Beckman Coulter) cell counter set to analyze 50 images with cell parameters set at minimum diameter 5 µm, maximum diameter 50 µm. The culture supernatants form these samples were used to obtain off-line reference data for glucose, lactate, glutamine, glutamate, and ammonia concentrations using the CuBiAn HT270 automated biochemistry analyzer (OptoCell).

Samples of the culture supernatants were also frozen at −80°C until completion of all bioreactor runs. These samples were then shipped on dry ice to Metabolon for analysis by Ultrahigh Performance Liquid Chromatography-Tandem Mass Spectroscopy (UPLC-MS). Briefly, samples were prepared by Metabolon using an automated MicroLab STAR system (Hamilton). Several recovery standards were added prior to the first step in the extraction process for QC purposes. Samples were extracted with methanol under vigorous shaking for 2 min using a GenoGrinder 2000 (Glen Mills) to precipitate protein and dissociate small molecules bound to protein or trapped in the precipitated protein matrix. This was followed by centrifugation to recover chemically diverse metabolites. The resulting extracts were divided into five fractions: two for analysis by two separate reverse phase (RP)/UPLC-MS/MS methods using positive ion mode electrospray ionization (ESI), one for analysis by RP/UPLC-MS/MS using negative ion mode ESI, one for analysis by HILIC/UPLC-MS/MS using negative ion mode ESI, and one reserved for backup. Samples were placed briefly on a TurboVap^®^ (Zymark) to remove the organic solvent. The sample extracts were stored overnight under nitrogen before preparation for UPLC-MS analysis. Metabolon’s proprietary software was used to match ions to in-house library of standards for metabolite identification and quantitation by peak area integration. Analyte concentrations were provided as normalized data in arbitrary units.

### Raman Spectroscopy Data Acquisition

Raman spectra were collected *in situ* using the RamanRxn2™ (Kaiser Optical systems, Inc.) analyzer coupled with four stainless-steel top mounted immersion probes fitted with standard adapters. The Raman probes were sterilized by autoclaving prior to being manually inserted into the BioBLU bioreactor. A laser excitation wavelength of 785 nm with power of 400 mW at the source resulting in approximately 275 mW of power output at the probe tip was used to generate Raman spectra from the cell cultures. Cosmic ray removal and dark spectrum subtraction were implemented using the iCRaman 4.1 spectral acquisition software. Acquisition was started approximately 16 h before inoculation of the cell culture and spectra were collected from each bioreactor vessel every hour throughout the course of culture using a 12.5 min spectral collection interval with 75 scans and an exposure of 10 s per scan.

### Raman Spectroscopy Chemometric Model Development

Raw time stamped spectra generated from triplicate runs of four donor T-cell cultures were aligned against daily sampling times for off-line metabolite measurements. Raman spectra were trimmed to keep only the relevant ranges of the spectrum (C-H range 3100—2,750 cm^−1^, and fingerprint 1850—250 cm^−1^). As commonly required for turbid biological media, baseline features were eliminated by calculating a first-order spectral derivative (moving window of 15 cm^−1^), and subsequent standard normal variate (SNV) standardization. Since distinct peaks for the metabolites of interest are typically not available in low concentration biological media, univariate regression did not apply. Instead, the target parameters were calibrated by multivariate regression with the Projection to Latent Structures approach (PLS1). A number of relevant features were selected visually with the help of the Variance Importance on the Projection ranking for each of the target parameters individually. In order to unbias the set of training samples for glucose and lactate models, all samples with zero concentration were omitted for the respective regression. Hotelling’s *T*^2^ criterion was used to exclude outliers from the respective models. All regressions were performed in cross-validation mode, leaving out the spectra from one donor type at a time (“leave-1 donor-out”). The selection of the optimum number of latent factors for each model was effectuated from the root mean squared error of cross-validation (RMSECV) *versus* Rank plot, using a rank as low as possible to reach an acceptable RMSECV. Chemometric model development was developed using SIMCA 13.

### Untargeted Univariate Raman Model Development

Algorithms for signal processing and data analysis were developed in Matlab (The MathWorks). All Raman spectra obtained from each bioreactor run were collated in a time-series. Each spectrum was smoothed and baseline correction was applied using the msbackadj function in Matlab to highlight the smallest peaks detectable in the spectra and emphasize any change in peak magnitude over time (Figures 3A–D). In order to compensate for systematic intensity biases between runs, baseline-corrected Raman intensities were normalized between the average value measured at three wavenumbers with constant low intensity (324, 564, and 1,483 cm^−1^) over time, and the average value at three wavenumbers (292, 295, and 2,926 cm^−1^) with constant high intensity (Figure 3F). Further normalization to *t* = 0 was applied by subtracting the average of the first two Raman spectra of the series to the rest of the series (Figure 3E). The identification of potential univariate Raman peaks matching the patterns of off-line measurements (glucose, lactate, glutamine, glutamate, cell concentration, cell viability) was achieved by correlation analysis of Raman intensities with off-line measurements. For each wavenumber, the 1-h resolution intensity time-series was discretized by averaging the 12 measurements recorded prior to the time of sampling the culture medium for off-line analysis. The collection of matching off-line measurements was also collated in the same order as the Raman series. Pairwise correlation analysis was performed by standardizing the intensity time-series at each wavenumber (using SNV normalization) and measuring its correlation coefficients *R* with the similarly standardized time-series of the off-line parameter of interest. The pairs with highest correlation (either positive or negative) were identified as surrogate univariate Raman markers whose patterns over time best matched those of off-line measurements.

### Datasets and Data Analysis

The same number of measurements was performed for all bioreactors and runs throughout the study. CuBiAn bioanalyzer data consisted of daily measurements for glucose, lactate, glutamine, glutamate and ammonia collected over a 10 days period. Cell concentration and viability data were also collected daily over 10 days using a Vi-Cell platform. LC-MS was used to measure the concentration of glucose, lactate, glutamine and glutamate daily for the first 9 days of the runs. The experiments were kept running for 12 days in total, during which Raman spectroscopy was performed hourly in all bioreactors. Where data was not available (1.6% of total data), data was either replaced by the average of the preceding and succeeding values (0.3% of total data), or by the preceding value when it was evident that the time series had plateaued (always when the lower limit-of-detection had been reached following analyte depletion, 1.3% of total data). All datasets were subjected to pairwise comparison using correlation analysis to quantify the linear relationship between variable pairs. For each analytical platform, all measurements from all donors and all run where first collated into a single dataset. Unless indicated otherwise, to compensate for the different scales between datasets, each variable from each dataset was auto-scaled using SNV standardization, and correlation analysis was performed between pairs of variables from different datasets. Agreement between such pairs was reported as the square of the correlation coefficient, or *R^2^*.

## Results

### Autologous Immunotherapy Model

**Figure 1:**
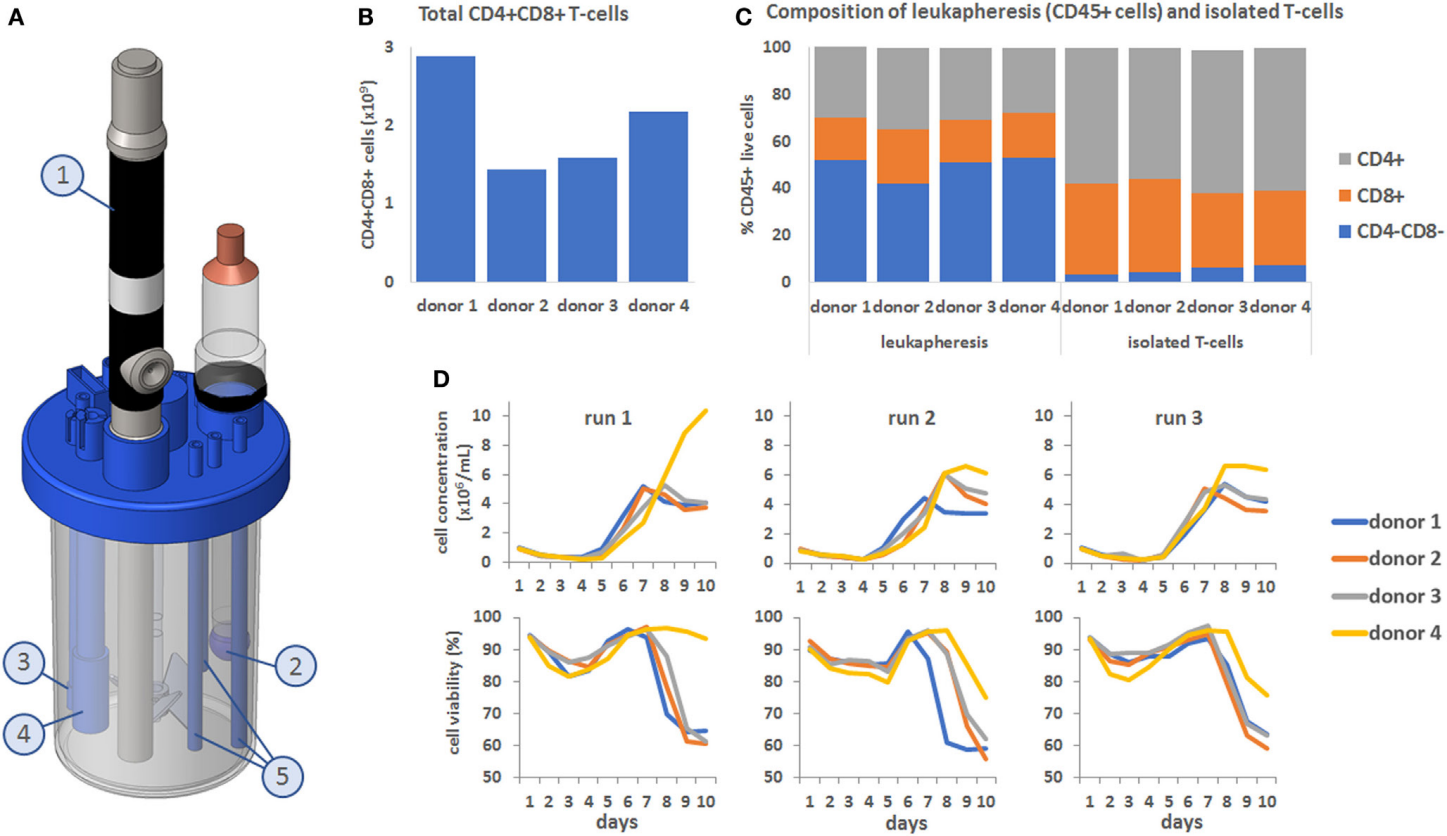
**(A)** Computer-assisted design image showing the setup of the Eppendorf BioBLU 300 mL single-use stirred tank bioreactor with Raman spectroscopy probe (1, shortened for visualization purpose), pH probe (2), temperature probe (3), dissolved oxygen probe (4) and fluid addition and sampling lines (5). **(B)** Donor-to-donor variability, expressed in total number of CD4+ CD8+ cells in the leukapheresis material. **(C)** CD4/CD8 composition of the CD45+ cells from the leukapheresis samples before T-cell isolation (first four bars on the left) and after T-cell selection (last 4 bars on the right). **(D)** Top row: cell concentration in the bioreactors over time, for all four donors and across all three process runs. Bottom row: corresponding cell viability curves.

All T-cell culture were maintained for 12 days in a BioBLU 300 mL single-use bioreactor containing a Raman spectroscopy probe (Figures 1A1) and sensors for pH (Figures 1A2), temperature (Figures 1A3), and DO (Figures 1A4). The processes were run for 12 days with a final feed on day 7 to examine cell behavior in response to depleted nutrients. To demonstrate the variability commonly encountered with primary cell material, T-cell counts were performed on leukapheresis material from four independent donors (Figure 1B) and counts of the CD4 and CD8 population made before and after T-cell isolation (Figure 1C). This showed that total T-cell counts in the incoming material varied between donors from 1.42 × 10^9^ to 2.89 × 10^9^ total T-cells. Variation in the number of CD4 and CD8 positive T-cells was high in the original leukapheresis samples but was reduced post T-cell isolation. However, the population of CD8 positive cells post isolation varied between 32 (donor 4) to 40% (donor 2). This isolated T-cell material from all 4 donors was then used to perform three independent process runs (*n* = 12), with cell behavior monitored through daily measurements of cell concentration and viability (Figure 1D). During the first process run, the samples from all four donors initially underwent a lag phase in growth lasting approximately 5 days as the cell recovered and adapted to the bioreactor environment. This was followed by a growth phase which occurred in a donor-dependant manner, with difference in the time at which the cells enter proliferation, their proliferation rates and their overall proliferative capacity. Interestingly in this bioprocess run, donor 4 entered proliferation later than the other donors but showed much higher proliferative capacity. In comparison, donors 1–3 stopped proliferating by day 7 and became quiescent. In bioprocess runs 2 and 3, similar trends were seen; however, the cells from donor 4 performed in a comparable manner to the other donors but still reached marginally higher overall cell numbers. For almost all process runs, viability of cells started to decrease after day 7. These results demonstrate the sometimes unpredictable nature of cell therapy processing with variability in the cellular material and in the behavior of cells between donors and within donors over different process runs.

### Chemometric Modeling

**Figure 2:**
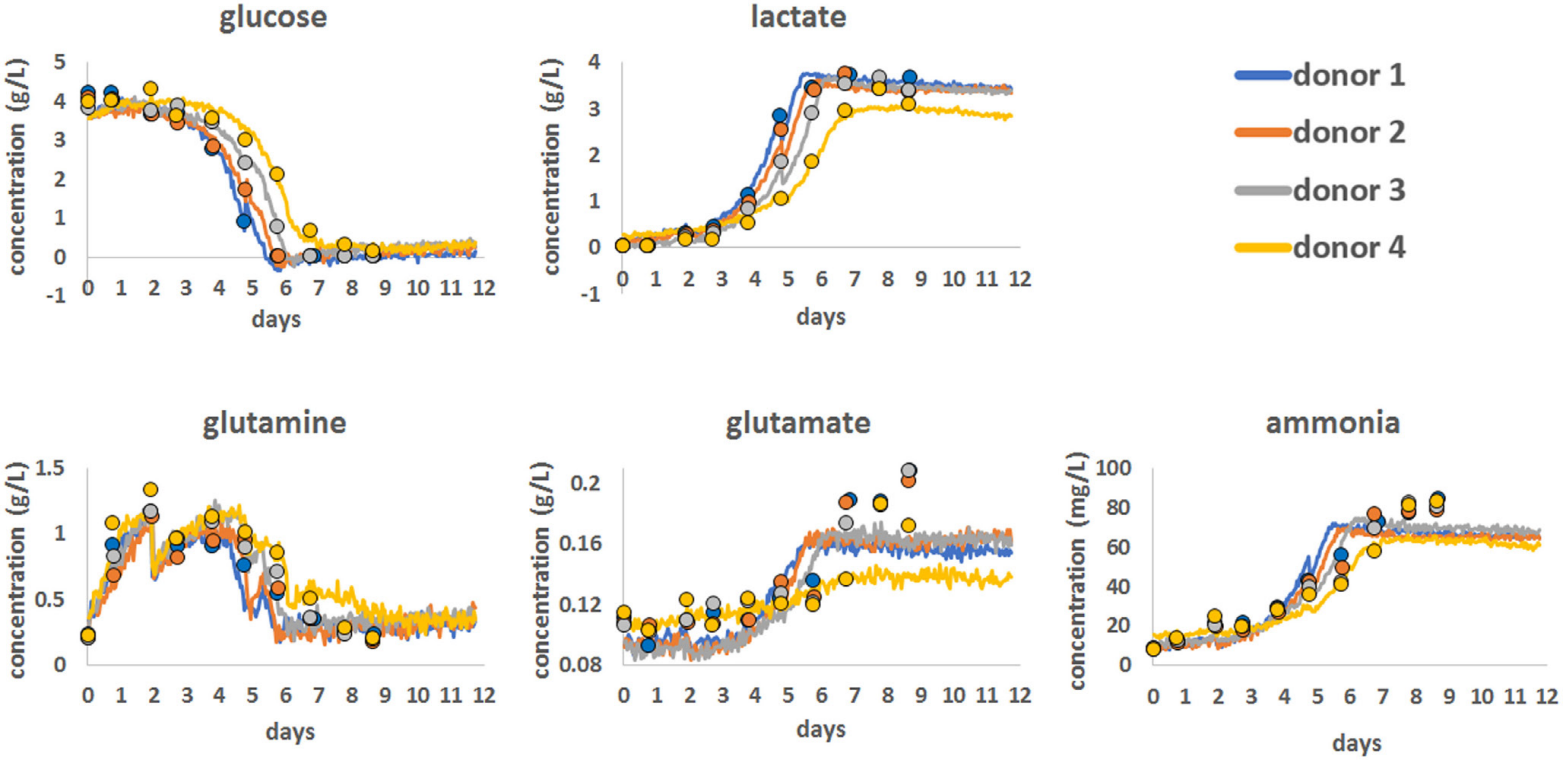
Chemometric models for glucose, lactate, glutamine, glutamate, and ammonia showing the correlation between reference bioanalyzer measurements (closed circles) collected every 24 h and continuous Raman chemometric analysis. Data for a single bioprocess run for all four donors are shown.

To demonstrate the use of Raman spectroscopy as a potential PAT tool to monitor the consumption of nutrients and the production of metabolites, a series of chemometric models were developed (Figure 2). These were produced using Raman spectroscopic data from all the bioprocess runs with 12.5 min of Raman data acquisition per hour for each bioreactor, for all 12 days. The statistical approach for Raman model development is detailed in the Section “Materials and Methods” and involved spectral pre-processing following by advanced statistical modeling using a PLS1 approach.

Using this approach, Chemometric models were successfully developed for glucose, lactate, glutamine, glutamate, and ammonia using reference data generated on a CuBiAn bioanalyzer to train the models. Best model fits were ranked based on the correlation between off-line data and model output as follow: glucose (*R* = 0.987), lactate (*R* = 0.986), ammonia (*R* = 0.936), glutamine (*R* = 0.922), and glutamate (*R* = 0.829). The chemometric models for glucose, lactate, and glutamine correlated well with the reference data and accurately predicted the consumption of nutrients (glucose and glutamine) and the production of lactate as cell metabolism and proliferation rates increased from day 5. For ammonia, the Raman models showed good correlation with the reference data at lower concentration but became less correlated above ~60 mg/mL. The model for glutamate showed the weakest correlation with only the general trend from low to high glutamate concentrations been measured and a loss of correlation above 0.16 g/L. Overall the Raman chemometric models tracked the reference measurements with a good degree of correlation and showed the potential for this technology as a PAT sensor.

### Univariate Raman Approach

**Figure 3:**
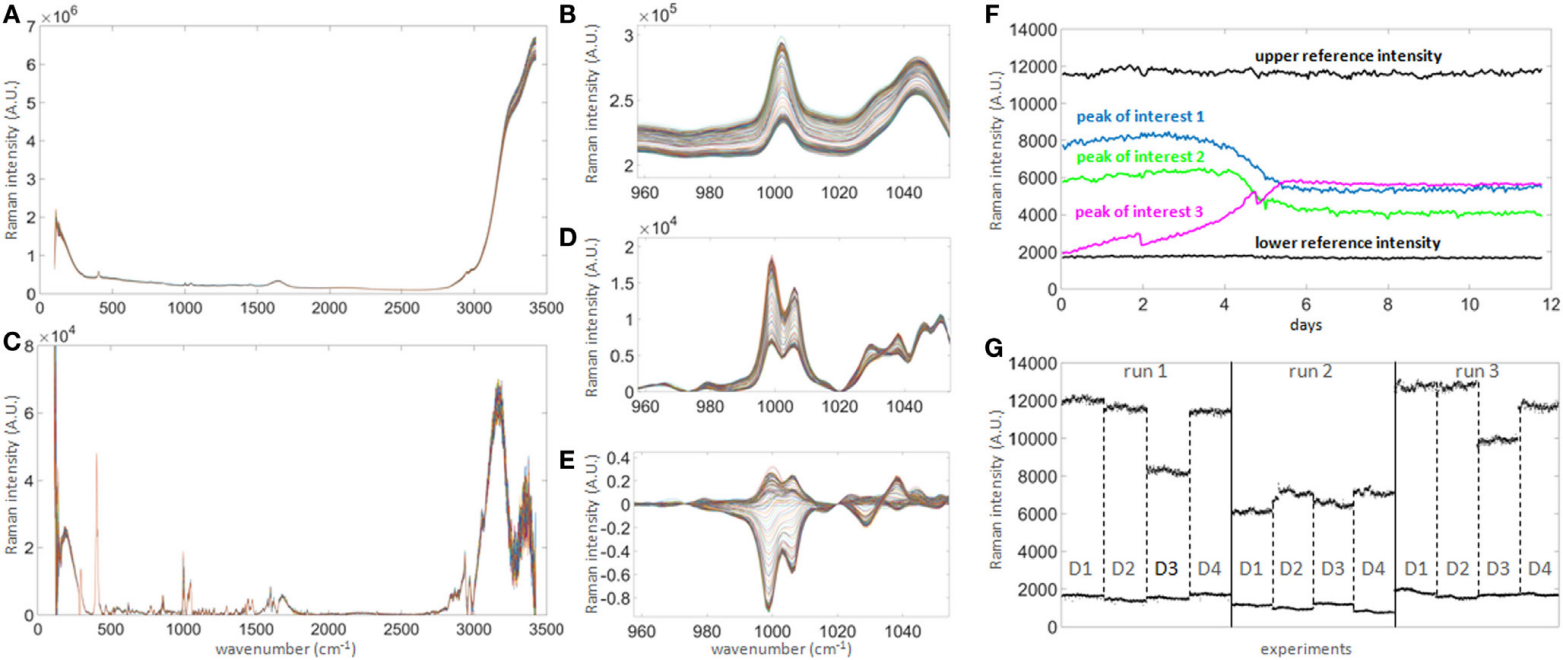
Raman signal processing for untargeted univariate data analysis. **(A)** 300 raw spectra collected hourly over 12 days in one bioreactor. **(B)** Magnified spectral region between wavenumbers 960 and 1,050, showing clear shift of signal baseline over time. The shift over time is of similar magnitude as most prominent peaks and hence needs to be corrected in order to quantify genuine changes of Raman intensity, such as seen in the subtle non-parallel patterns on the left-hand side of the right peak. **(C)** Baseline-corrected spectra. The correction highlights the potential peaks of interest. **(D)** Same region as in **(B)** after correction, demonstrating that the spectra are significantly re-aligned. **(E)** Same as **(D)** after subtracting the averaged first two spectra, in order to normalize the signals to *t* = 0 and highlighting true signal fluctuations over time. **(F)** Example of time series for three peaks of interest (colored) showing patterns consistent with expected biochemical changes in the culture medium. As the intensity scale is not calibrated to a standard, two reference intensities consisting of the averages of three peaks with either constant high (at 292, 295, and 2,926 cm^−1^) or low (at 324, 564, and 1,483 cm^−1^) intensities over time (labeled “upper reference intensity” and “lower reference intensity,” respectively, the bold black lines showing the averages of both groups) were selected to perform internal signal normalization. This approach was used to normalize all time series between runs. **(G)** Comparison of reference time series between all runs and all donors (labeled D1–D4). This graph illustrates the unpredictable extensive probe-to-probe variability observed with this technology in this context, whether technical or biological, and the need for signal calibration or normalization to obtain comparable datasets between different bioreactors and experiments.

The identification of potential univariate markers in the Raman time series was investigated as an alternative to chemometric modeling and as an online calibration-free methodology. Correlation of Raman time series with raw off-line measurements did not initially identify a large number of potential Raman markers. Although the position of the Raman peaks remained stable over 12 days of signal acquisition (Figure 3A), they were small and of similar magnitude to the shift of intensities over time (Figure 3B). Standard baseline correction was therefore applied (Figure 3D) but did not yield the detection of any more strongly correlated Raman markers due to the unpredictable fluctuation of the dynamic range between probes and runs (Figure 3G). However, normalization between the means of two sets of peaks with constant low and high intensities during run acquisition (Figure 3F, dark horizontal traces), followed by normalization to *t* = 0 (Figure 3E), greatly increased the number of well correlated Raman markers with all off-line measurements tested (with the exception of viability). These measures could be implemented during real-time signal acquisition, potentially removing the need for calibration.

### Correlation between Chemometric and Univariate Raman Approaches with Off-Line Measurements

Prior to performing correlation analysis, the errors associated with the off-line measurements were evaluated as the ratios of the standard deviation of triplicate measurements divided by their means, or coefficient of variations (CV). For the LC-MS platform, the CV for glucose, lactate, glutamine and glutamate were found to be 1.4, 3.5, 1.9, and 2.6%, respectively. On the CuBiAn bioanalyzer platform, the corresponding CV were 0.9, 1.1, 1.2, and 3.7%, respectively, with the additional ammonia CV = 1.7%. For the ViCell, cell concentration had a 4.0% CV, and cell viability a 1.3% CV. Therefore, all trends observed in the subsequent analysis were found to be significant.

**Figure 4:**
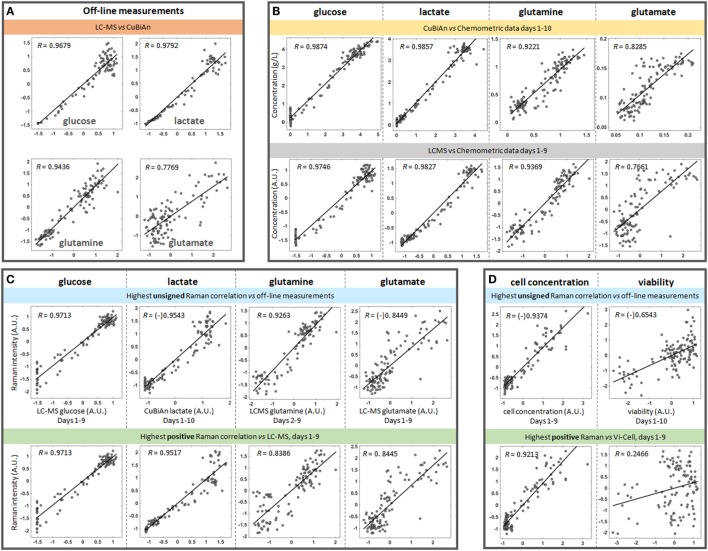
Representative correlation plots between datasets. All off-line measurements from all donors and all runs were used in these plots, with an average of 108 points used per comparison. Unless indicated otherwise on the axes, the pairwise datasets were auto-scaled using standardization and expressed in arbitrary units (A.U.) to make the scales comparable. When the data is negatively correlated in the original dataset, a “(-)” symbol is placed before the value of the correlation to indicate the original sign of such correlation. **(A)** CuBiAn *versus* LC-MS correlations highlight the variability between the two off-line platforms. **(B)** Correlations between chemometric models and CuBiAn data used to build the models. **(C)** Best correlations observed between blind univariate Raman markers and off-line measurements for each nutrient and metabolite (top row) and best positive correlations between LC-MS measurements (bottom row). **(D)** Cell concentration and viability correlated to the univariate Raman models.

In order to ensure that both the chemometric and univariate Raman approaches had the ability to identify Raman peaks which correlated with reliable reference datasets, pairwise correlation analyzes were first performed to test the agreement of off-line metabolite measurements from a bioanalyzer and a LC-MS system for glucose, lactate, glutamine, and glutamate (Figure 4A). This analysis showed good correlation between the datasets for glucose (*R* = 0.9679), lactate (*R* = 0.9792), and glutamine (*R* = 0.9436) indicating that data from either system could be used for univariate model development. However, there was weaker correlation for the reference datasets for glutamate (*R* = 0.7769) indicating that development of good univariate models could be more challenging. These correlations and the spread of the points around the best fitting line provided reference information from which to compare the performances of the chemometric and univariate Raman approaches.

Next a correlation analysis was performed between the chemometric models and the dataset from the bioanalyzer and LCMS system (Figure 4B). As expected the highest correlations were observed between the chemometric models and the CuBiAn data for glucose (*R* = 0.9874) and lactate (*R* = 0.9857) as these were reference measurements used to train the chemometric models (Figure 4B, top row). High correlations were also observed between the chemometric models for glucose and lactate and the LC-MS data (Figure 4B, bottom row). This demonstrates the robustness and relevance of the chemometric approach for these analytes. The chemometric model for glutamine correlated well with both CuBiAn and LC-MS datasets (*R* = 0.9221 and 0.9369, respectively), whereas glutamate exhibited weaker correlation and could only be useful for general trend analysis rather than more accurate quantitative measurement.

To further test the univariate approach, correlation analysis was performed against all the reference datasets (Figure 4C). Two approaches were used. The first was an unsigned approach which correlated the univariate Raman spectra against the reference data, i.e., irrespective of whether the correlation was positive or negative (Figure 4C, top row). This was done to identify the overall best surrogate markers from the Raman spectra. The second approach was performed using only positive correlations (Figure 4C, bottom row), as these may relate to components of the spectral signature of each analyte. The unsigned approach consistently yielded the strongest correlations, and they were only slightly lower than or comparable to those from the chemometric models for glucose, lactate, and glutamine (*R* = 0.9716 *vs*. 0.9874 for glucose, 0.9543 *vs*. 0.9857 for lactate, and 0.9263 *vs*. 0.9221 for glutamine). As expected from previous correlation measurements, glutamate exhibited a lower correlation (*R* = 0.844 for either univariate Raman approaches), but slightly higher than the chemometric model. However, the trend from low to high concentrations remained highly visible. This analysis indicates that the univariate models could be used to track nutrient consumption and metabolite production to a similar level of accuracy as the chemometric models. Interestingly, as shown in Figure 4D, a strong correlation was also observed between unsigned univariate Raman (*R* = 0.9374) and positive univariate Raman (*R* = 0.9213) with cell concentration. This indicates that the univariate Raman approach could be also used to monitor changes cell number over time within the bioreactor system. A weaker correlation was found for cell viability (*R* = 0.6543) meaning the application of univariate modeling for tracking cell viability is probably limited to trend analysis of viability within the bioreactor.

All pairwise comparisons including results for ammonia are summarized in Table 1. Day 1 data was sometimes omitted in the pairwise comparisons as they resulted in poorer correlations. Bold numbers indicate the strongest correlation among all pairwise analyses for each analyte. Most of the strongest correlations were for the chemometric models *versus* CuBiAn data, this was expected as these were the reference data used to calibrate the models. The underlined numbers indicate the highest correlations obtained using the blind univariate Raman approach. Best correlations were exclusively observed with the LC-MS dataset, suggesting that LC-MS may be more robust system to generate data for Raman model development. Ammonia showed similar correlation irrespective of whether chemometric or univariate Raman approaches were used. Table 2 shows the datasets best correlated with univariate Raman markers. With the exception of glutamate and cell viability, high correlations were achieved for all parameters, indicating that the univariate Raman approach can yield good inferential markers to track these variables. The mixture of positive and negative correlations observed to achieve such high values suggests that some of the Raman markers may be surrogate markers. This is highlighted in Table 3 which summarizes all best correlations either positive or negative (in case the latter was stronger than the former), with reference data from both the CuBiAn and LC-MS systems.

**Table 1:** Correlation between all standardized metabolite datasets investigated in this study.

Data source	Pairwise conditions	Days	*R* of standardized data				
			Glucose	Lactate	Glutamine	Glutamate	Ammonia
Off-line	CuBiAn/LC-MS	1–9	0.968	0.979	0.944	0.777	N/A

Chemometrics	Chemometrics/LC-MS	1–9	0.975	0.983	**0.937**	0.766	N/A
	Chemometrics/CuBiAn	1–10	**0.987**	**0.986**	0.922	0.829	**0.936**

Raman univariate predictors	Best positively correlated/LCMS	1–9	0.971	0.952	0.839	**0.845**	N/A
	Best positively correlated/LCMS	2–9	0.970	0.944	0.926	0.835	N/A
	Best positively correlated/CuBiAn	1–10	0.958	0.673	0.654	0.177	0.935
	Best positively correlated/CuBiAn	2–10	0.956	0.701	0.899	0.173	0.931

	Overall means		0.97	0.89	0.87	0.63	0.93

*The datasets consisted of off-line CuBiAn bioanalyzer data for glucose, lactate, glutamine, glutamate, and ammonia collected daily over 10 days*.

*Bold numbers indicate the strongest correlation among all pairwise analyses for each analyte. The underlines numbers indicate the highest correlations obtained using the blind univariate approach*.

**Table 2:** Datasets best correlated with univariate Raman markers.

Variable	Off-line dataset	*R*	Sign of correlation
Glucose	LC-MS day 1–9	0.971	Positive
Lactate	CuBiAn day 1–10	0.954	Negative
Glutamine	LC-MS day 2–9	0.926	Positive
Glutamate	LC-MS day 1–9	0.845	Negative
Ammonia	CuBiAn day 1–10	0.935	Positive
Cell concentration	Vi-Cell day 1–9	0.937	Negative
Viability	Vi-Cell day 1–10	0.654	Negative

**Table 3:** Overall best signed and unsigned correlation results between Raman *vs*. CuBiAn, and Raman *vs*. LC-MS measurements.

	Off-line measurements	Highest absolute correlation *R*	Highest positive correlation *R*
Raman *vs*. CuBiAn (days 1–10)	Glucose	0.9576	N/A
	Lactate	−0.9543	0.9502
	Glutamine	0.8465	N/A
	Glutamate	−0.8621	0.836
	Ammonia	0.9352	N/A
	Cell concentration*	−0.9308	0.905
	Viability*	−0.6543	0.6111

Raman *vs*. LC-MS (days 1–9)	Glucose	N/A	0.9713
	Lactate	N/A	0.9517
	Glutamine	N/A	0.8386
	Glutamate	−0.8449	0.8445
	Ammonia	N/A	N/A
	Cell concentration*	−0.9374	0.9213
	Viability*	−0.5977	0.2466

*Cell concentration and viability measured by ViCell.

### Application of Raman Spectroscopy for Time Course Studies and Real-time Monitoring

**Figure 5:**
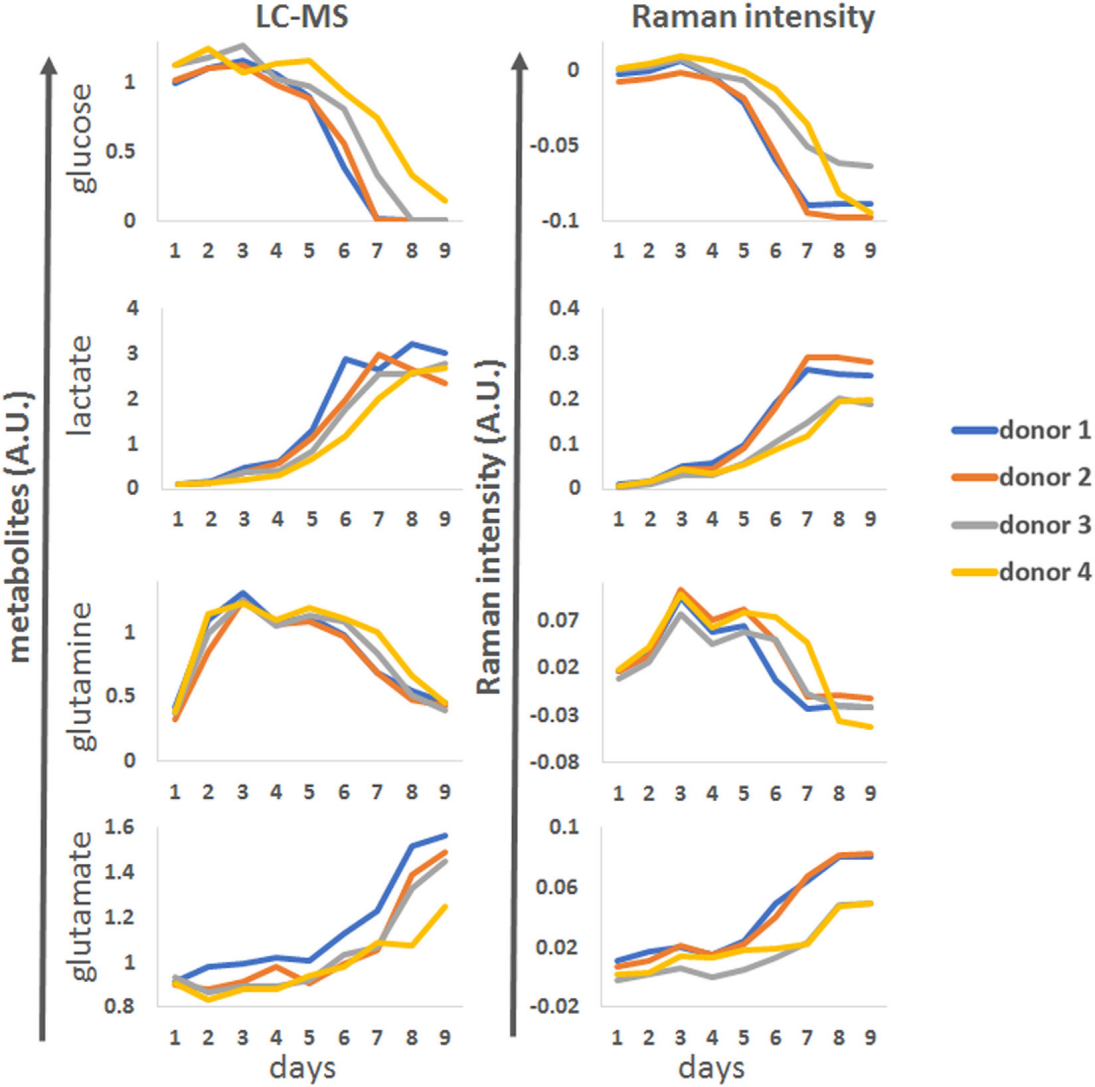
Representative examples of glucose, lactate, glutamine and glutamate time course patterns observed by LC-MS (right column, arbitrary units A.U.) for four donors during process run 1, compared to matching univariate Raman markers.

To demonstrate the application of the univariate analysis for biomarker monitoring, we compared the LC-MS reference data with best matching profiles from the discretized univariate Raman time series (Figure 5). Overall, there was good agreement between the Raman models and the LC-MS reference data for all the analytes. The univariate models could accurately predict changes in marker expression including the donor-dependant decrease in glucose around day 5, the increase in lactate levels which accelerated from day 4 and the multiphasic expression of glutamine. As expected based on the correlation analysis, the model for glutamate was less robust but the general trend for this analyte could be followed.

**Figure 6:**
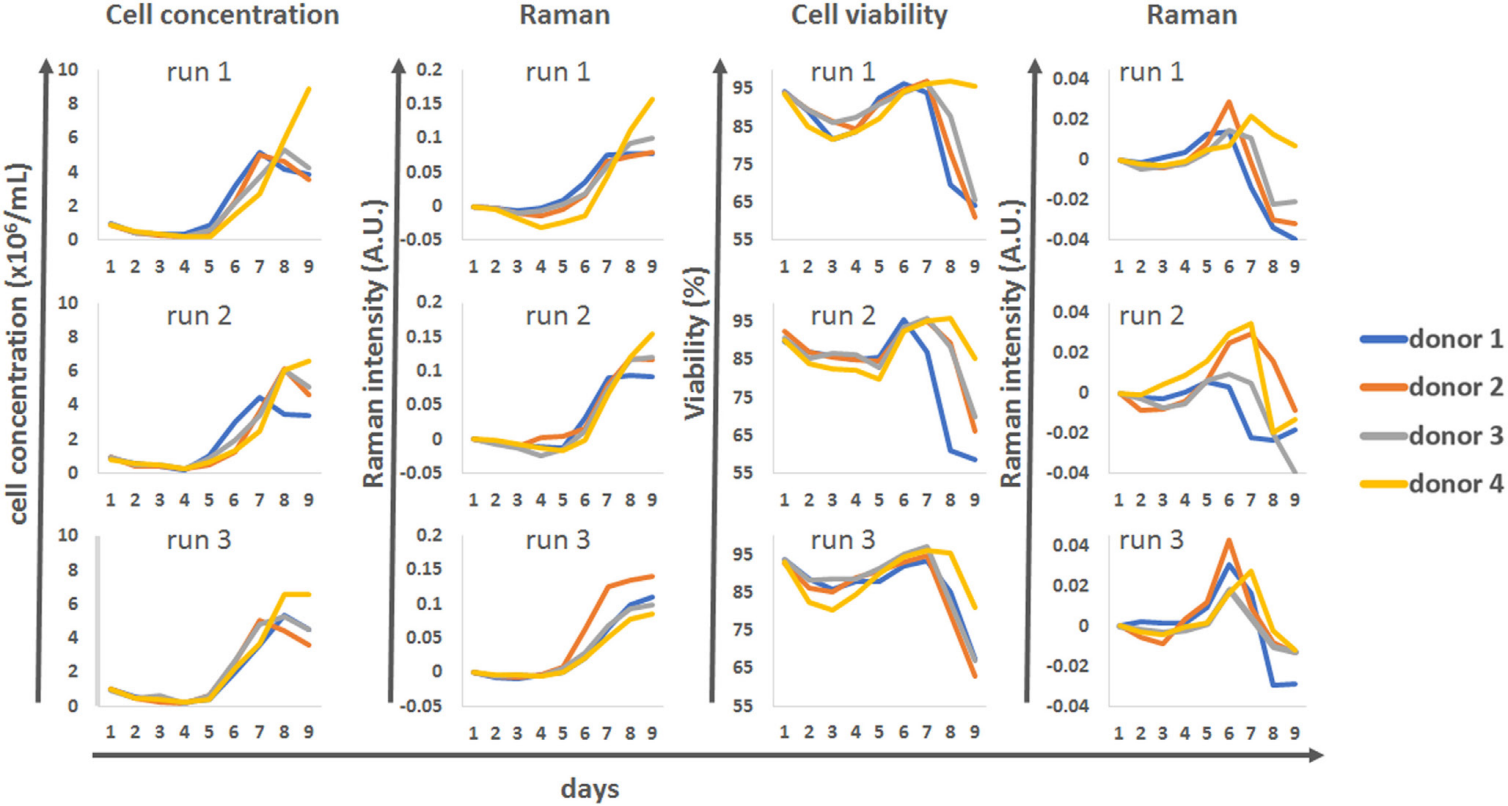
Discrete time course data for cell concentration and viability, for all donors and all runs, compared to matching univariate Raman modeling.

The univariate Raman models were compared against the reference datasets for cell concentration and viability for all four donors and the three production runs (Figure 6). This analysis showed that for runs 1 and 2 there was very good agreement between the reference cell concentrations measured using an automated cell counter and the Raman model. The Raman models could be used to track the cells as they progressed from their initial static phase toward a proliferative phenotype around day 5. The model also predicted the donor-dependant transition of some of the culture to a quiescent phenotype from day 7. Model correlation for run 3 was not as strong, but this may be due to the level of variability associated with the reference cell counts. Overall this data showed that the univariate models could be used as surrogate markers to track cell behavior within the bioreactor system.

A lower level of agreement was observed between off-line viability measurements and Raman time series. However, there were interesting correlations between these patterns, with the Raman marker consistently peaking 1 day earlier than the off-line viability measure. The overall times at which the donors stopped proliferating was also well represented with the Raman “viability” marker, with donor 1 reaching its plateau phase earlier than the other donors in runs 1 and 2 but not run 3.

**Figure 7:**
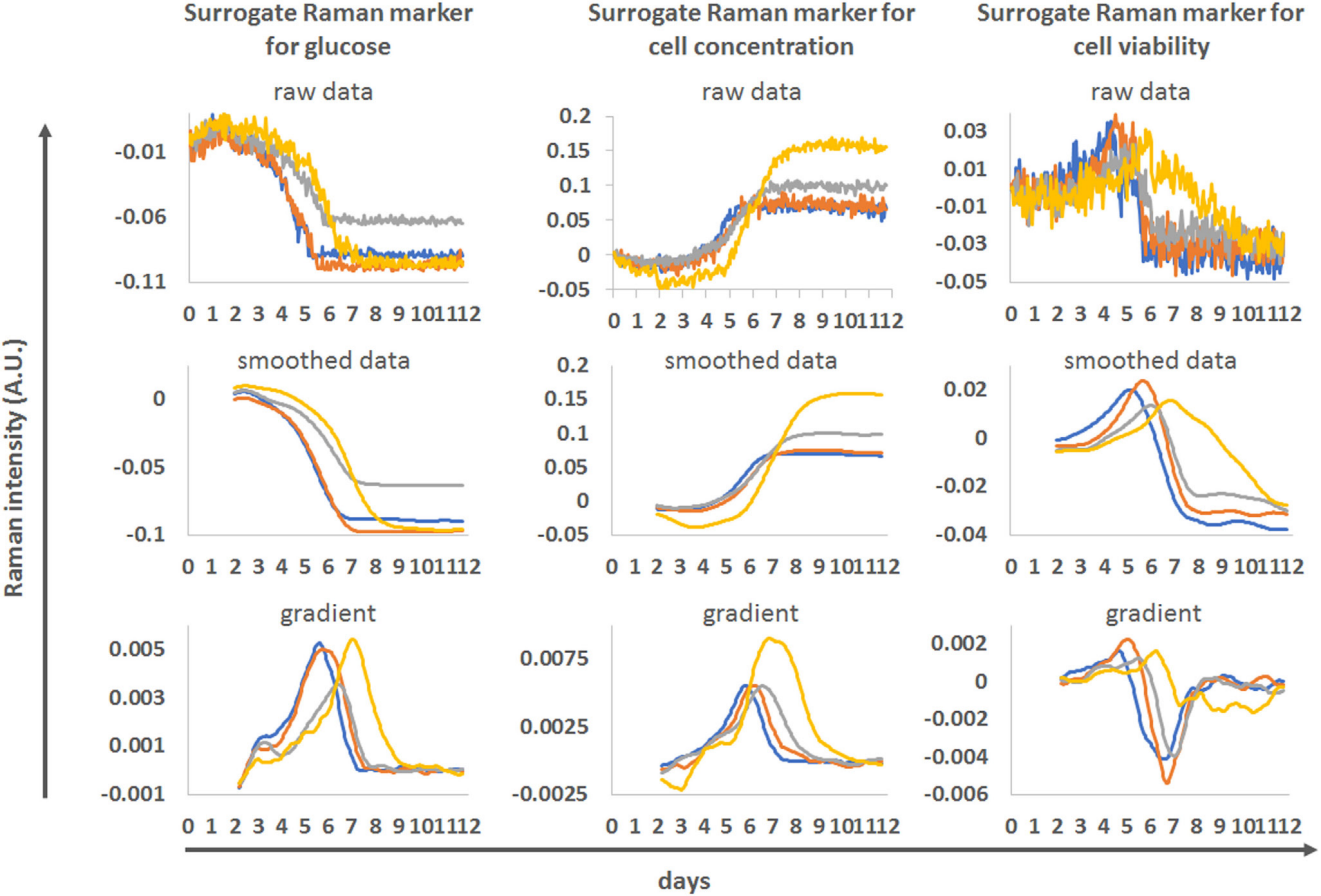
Example of real-time monitoring of process runs using the blind univariate Raman approach as surrogate markers for glucose (left), cell concentration (middle) and cell viability (right). The four donors from run 1 were used. Top—unfiltered Raman time series for these markers. Middle—same data after application of a 24 h moving averaging filter. Bottom—gradient of the smoothed Raman time series.

The actual value of online Raman spectroscopy is in its potential to monitor complex bioprocesses in real-time. Some of the Raman markers (markers for glucose, cell concentration, viability) identified through the blind univariate approach described in this study are presented in their real-time format in Figure 7. Unfiltered Raman time series shows the actual level of noise present in the datasets. For the real-time monitoring of glucose and cell concentration, the level of noise in the Raman models is low, allowing detailed monitoring of glucose consumption and changes in cell behavior. For the viability measurements, the noise level is high but overall trends in viability can be monitored. To smooth the Raman data, we applied a 24-h moving filter (i.e., at any given time, the Raman value was the average of the 24 previous measurements) the noise was removed and similarities or differences between the donors where highlighted. This type of running filter could be applied in real-time. The derivation of the smoothed curves highlights the rate of changes in marker intensities, producing clear peaks at different time points between donors. These could also be used to track the progression of each culture and inform process actions such as initiating feeds or indicating time to harvest in real-time.

## Discussion

A wide range of bioreactor systems are used for cell therapy bioprocessing and many of them provide in-line sensors which can be used for routine real-time monitoring of physical parameters such as pH, dissolved oxygen and temperature. However, measuring biological and functional parameters relating to cell behavior within a culture system is much more complex. In this study, we applied in-line Raman spectroscopy as an optical sensor for real-time monitoring of T-cell behavior during immunotherapy bioprocessing. The primary aim was to demonstrate the use of Raman spectroscopy to monitor the consumption of nutrients (glucose, glutamine) and the production of markers associated with cell metabolism (lactic acid, glutamate, and ammonia). This is challenging due to the complexity of the culture environment in which the cells are maintained. Typical culture media, even when chemically defined, consists of over 40 components including inorganic salts, amino acids, sugars, vitamins, alpha-keto acids, and pH indicators such as phenol red. Therefore, any sensors applied for functional monitoring of cell behavior must be able to make precise measurements of target nutrients and metabolites without interference from the other multiple components present. Furthermore, because cell therapy producers are increasingly closing their manufacturing processes, any sensor used for bioprocess monitoring could need to function optimally for several weeks.

While other studies have shown the successful application of Raman spectroscopy for bioprocess monitoring, this has typically been performed in relatively simple culture environments and using well characterized cell lines that follow predictable growth profiles. For example, CHO cell cultures consistently show exponential cell expansion immediately following seeding into the bioreactor system (12). As a result, Raman models can be developed using a small number of bioprocess runs with calibration often achieved by spiking known concentrations of the analytes of interest into the media to create standard curves to support Raman model development (19). In this study, the primary T-cells derived from different donors show multiphasic growth profiles. Initially, there is a static period lasting approximately 5 days as the cells recover and adapt to the culture environment, the cells then enter a rapid proliferative state lasting 3–4 days before some cultures enter a quiescent phase. The initial static period is consistent between donors but after 5 days the growth profiles become divergent with proliferation rates, overall population doublings and viability changing in a donor-dependent manner. This divergence makes real-time monitoring more important, particularly if components have to be maintained within tight specifications to control cell quality. The complexity and composition of the culture environment can also affect the performance of Raman optical sensors. The media formulation used to support cell growth in this study was a chemically defined formulation. However, it was supplemented with a mix of cytokines (interleukin-2), a polymeric nanomatrix conjugated to monoclonal antibodies (CD3 and CD28) and human serum, all of which can have lot-to-lot variability. In addition, even though the basal media was a defined formulation, the exact composition is proprietary and not provided by the supplier. This is a common situation faced by cell therapy producers. Unlike biopharmaceutical production where at least 70% of recombinant proteins are produced from CHO cells (20), cell therapies are developed using a wide range of different cell types. As a result, there is now an expanding market for proprietary commercial media formulations. This level of media complexity and lack of information relating to media composition makes Raman model development and data analysis more challenging. It may also limit the applicability of Raman spectroscopy during early process development when media supplements and basal media formulations are still being developed. Instead, Raman spectroscopy may have most benefit for cell therapy monitoring once a basic process is defined to allow fine tuning during the ongoing optimization process.

In this study, the complexity of the culture system led to the production of Raman data that consisted of weak overlapping peaks from all components in the media, making it difficult to identify and quantify spectral features for individual target analytes. This was expected as it has been reported as a challenge even in simpler culture systems (12, 13, 17). Consequently, advanced chemometric approaches were required for spectral pre-processing and measurement of the analytes of interest. A combination of first-order spectral derivative, SNV standardization for baseline correction and a PLS1 approach was used to model the analytes of interest. The chemometric models were calibrated using reference measurements taken every 24 h on an off-line photometric bioanalyzer. Using this approach, the Raman model predictions matched the reference values for glucose, lactate, and glutamine with high a high degree of correlation across the concentration range of the reference datasets and throughout the whole process. The model used to predict the concentrations of ammonia showed good correlation over the lower concentration ranges but became less predictive above 60 mg/L. In comparison, the model for glutamate showed poor correlation with the reference measurement in this cell therapy system. This is likely due to the accuracy of the glutamate reference method produced on the bioanalyzer system that is toward the lower limits of its detection range. Importantly, the Raman models accurately modeled the depletion of glucose and glutamine from the media and increased production of lactate and ammonia, with these changes corresponding to the transition of the cells from a recovery phase (days 1–5) toward a proliferative state. This correlation between the in-line Raman models and the off-line reference measurements show the potential for real-time tracking of these key markers during cell therapy manufacture.

An interesting approach investigated in this study is the use of univariate Raman modeling for non-targeted analysis of the culture media. This approach involves relatively straightforward spectral pre-processing using baseline correction, internal normalization using reference peaks, and normalization of the data to *t* = 0, all of which can be implemented in real-time. Changes in the processed spectra can then be used to provide in-depth information about both cell behavior and the culture environment. As an initial application of this approach, each individual Raman wavenumber was analyzed and compared to reference measurements for glucose glutamine, glutamate, and lactate produced using both a bioanalyzer and LC-MS system. This enabled the identification of peaks with the strongest correlation to the reference measurements. These could be used to track the nutrient depletion (glucose and glutamine) and metabolite production (glutamate and lactate), to a comparable level of accuracy to the chemometric models. However, the advantage of this univariate approach is that these peaks can be used as surrogates to monitor processes changes without the need for the complex statistics required for chemometric model development. This univariate approach can also be used to correlate Raman peaks with other process parameters for which reference measurements are available. We showed how this can applied to accurately track cell concentration including cell proliferation rates and the overall proliferative capacity of the cells derived from difference donors. While not shown in this study, this univariate approach could also be used to identify peaks which change in a consistent way between individual bioreactor runs, thereby creating a process fingerprint. The use of fingerprinting for bioprocess monitoring has been reported at a holistic level using techniques such as refractive index sensors to track non-specific changes in the culture environment and establish acceptable ranges for process performance (21). These holistic approaches can be valuable for general monitoring of bioprocesses and could be used to highlight processes that are running suboptimally. The univariate Raman modeling approach presented here could potentially offer an even finer level of resolution for process fingerprinting. This is because 10s or 100s of individual peaks could be measured simultaneously in real-time and used to develop a detailed multivariate design space to track process performance and measure the level of consistency between process runs. As changes or refinements are made to a process over time, these same fingerprints could be used to help demonstrate comparability.

The ability to measure and track the expression of key markers during cell therapy bioprocessing opens up interesting opportunities for real-time monitoring and progress within the field toward feedback control during product manufacture. Other reports have shown that Raman spectroscopy can be used for closed loop control to improve biopharmaceutical protein production by monitoring parameters such as lactate concentration (22) or glucose consumption (23, 24). However, this is dependent upon the accuracy and robustness of the spectroscopic models used for tracking these key makers (25). While these types of real-time closed loop systems have not yet been applied to cell therapy manufacture, they could provide the level of control required to manage the variability that currently resides in autologous manufacturing processes. In the shorter term, Raman spectroscopy could be used to inform the timings of key process decisions. For example, coordinating viral transduction with the transition of the cells to a proliferative or activated state. The data presented here shows how a combination of Raman analysis for cell number in combination with changes in nutrient consumption could be used to indicate the point where cells are entering proliferation and to time viral addition. This could have significant benefits for optimizing the use of key raw materials which have a large impact on the overall cost of goods for cell therapy manufacture.

Overall, the data presented in this study demonstrates the potential of using Raman spectroscopy to monitor the functional behavior of cells in real-time during cell therapy bioprocessing. To our knowledge, this is first report applying Raman technology to monitor the consumption of nutrients and the production of metabolites in a cell therapy model. We also believe this is the first report showing the potential for using univariate modeling for real-time non-targeted correlation analysis of viable cell concentration. The ability to have real-time measurements of these key parameters could provide immediate feedback on process performance and make Raman spectroscopy an attractive PAT system to improve future cell therapy manufacturing processes.

## Author Contributions

M-OB performed the data and statistical analysis, and developed the Raman univariate model that underpinned this study. M-OB also contributed substantially to the preparation of the manuscript and to the figures. DB was responsible for developing the immunotherapy model used in this study including novel bioprocessing steps. DB also analyzed and prepared the data used in Figure 1 and contributed to the preparation of the manuscript. EH was responsible for establishing the Raman spectroscopy approach used in this study. EH also contributed to the bioprocessing runs, collected and analyzed off-line samples and input in the preparation of the manuscript. DM was principal investigator for this study, derived the original concept for the work, and led the experimental studies. DM also made a significant contribution to the preparation of the manuscript and figures.

## Conflict of Interest Statement

The authors declare that the research was conducted in the absence of any commercial or financial relationships that could be construed as a potential conflict of interest. The reviewer DCW and the handling editor declared their shared affiliation.
